# Critical Review of Hofmeier: Upon the Value of Prophilactic Irrigation of the Uterus Immediately after Delivery

**Published:** 1881-12

**Authors:** P. W. Van Peyma


### Translations.

### Critical Review of Hofmeier: Upon the Value of Prophylactic Irrigation of the Uterus Immediately after Delivery.

Max Runge: Of Observations upon an Epidemic of Puerperal Fever at the Obstetrical Clinic of the Charity Hospital. H. Fehling: Upon the Practical Value and the Method of Disinfecting in Obstetrical Practice, and Breisky: Upon Local Intra-uterine Treatment of Puerperal Fever.

From the German by P. W. Van Peyma, M. D.

An active revival has occurred of the question of intra-uterine treatment immediately after birth, or during the parturient condition. Upon the meritorious pamphlet of Fitsch there followed, as is well known, a list of enthusiastic reports that placed the local intra-uterine treatment in the most favorable and least dangerous light. On the other hand, warning voices have in more recent times been raised that cautioned us against its too genneral use, and showed the indisputable objections, as developed in practice. This change of opinion must be looked at as an advance. Uterine irrigation has now passed into the hands of the general practitioner. These one-sided reports, according to which irrigation was possessed of great advantages, and was entirely harmless, are at fault in that they were indiscriminately and wrongly employed. Being used after every normal birth, in the slightest aberrations of the parturient condition, in cases of discolored lochia, they often did more harm than good. The practitioner felt no blame in these occasional ill results; on the contrary, he with right fell back upon these publications of the larger institutions in which irrigations were extolled as harmless, as important prophylactic, and as curative in puerperal fever. He thought he would have been inexcusable had he not made use of this means.

Soon, however, as well as later, attention was called to serious accidents and even deaths immediately after irrigation.

However, as these cases were not very numerous, they were attributed not to any inherent dangers, but to the faulty method employed. And it is only the later observations of Hofmeier, Runge, Fehling and Breisky that have placed the objections to intra-uterine treatment in their true light, and have limited its scope of applicability.

Hofmeier, in reviewing the prophylactic intra-uterine irrigation as practiced in Schweder's clinic, has pointed out that the health standard is lowered. Of 260 normally delivered women who were immediately subjected to irrigation, 16.1 per cent. became sick, while of 240 not thus treated only 8 per cent. suffered illness. In consequence of this showing, Hofmeier has laid aside all prophylactic intra-uterine irrigation after normal deliveries. Only in cases where morbid processes are occurring in the post-partum uterus, is the cavity of the uterus disinfected with a 5 per-cent. solution of carbolic acid.

Runge has shown in Gusseron's clinic that post-partum prophylactic uterine irrigations, which were employed experimentally in the beginning of a so-called puerperal fever epidemic, in reality promoted the spread of the septic poison. This consideration is of importance to the practitioner, for the reason that if the practice should become common, the same physician would often be constrained both to attend the patients and to perform the irrigation. Only where septic processes are taking place in the uterus does Runge allow irrigation. According to this principle, 1,500 cases were treated and only 39 died from septic poisoning.

Fehling, also, in his small and readable treatise, warns against too meddlesome treatment of the parturient. He also has had unsatisfactory experience with intra-uterine irrigations at the Stuttgart Hospital, and on this account has entirely discontinued them.

Breisky is very critical. He throws aside especially regular uterine irrigation and drainage, as he has observed threatening symptoms and spread of the infecting material, as a result of their employment. As the preferable form, he endorses the continuous irrigation of Schweder, which results in the washing away of the secretions and in a diminution of the temperature. In proof whereof he reports fifteen cases. In all these cases a special assistant, who was not allowed to attend in delivery, was entrusted with the operation. This fact, to say nothing of its inherent want of practicability, would exclude it from general private practice. In consequence of his experience he has done away with all uterine and vaginal irrigation, except where special morbid processes demand it. In normal cases he agrees with Spiegeberg that non-interference is the preferable course. Neither has Breisky been able to convince himself that the mortality of puerperal fever can in any great measure be lessened by a particularly active and regular prophylactic injection of the internal genital organs.

